# Adjuvant radiotherapy after radical cystectomy for muscle-invasive bladder cancer: A retrospective multicenter study

**DOI:** 10.1371/journal.pone.0174978

**Published:** 2017-04-06

**Authors:** Mathieu Orré, Igor Latorzeff, Aude Fléchon, Guilhem Roubaud, Véronique Brouste, Richard Gaston, Thierry Piéchaud, Pierre Richaud, Olivier Chapet, Paul Sargos

**Affiliations:** 1 Department of Radiotherapy, Institut Bergonié, Bordeaux,France; 2 Department of Radiotherapy, Groupe Oncorad Garonne, Clinique Pasteur, Toulouse, France; 3 Department of Medical Oncology, Centre Léon-Bérard, Lyon, France; 4 Department of Medical Oncology, Institut Bergonié, Bordeaux, France; 5 Clinical and Epidemiological Research Unit, Institut Bergonié, Bordeaux, France; 6 Department of Urology, Clinique Saint-Augustin, Bordeaux, France; 7 Department of Radiotherapy, CHU de Lyon-Sud, Lyon, France; University of British Columbia, CANADA

## Abstract

**Objectives:**

Radical cystectomy (RC) and pelvic lymph-node dissection (LND) is standard treatment for non-metastatic muscle-invasive urothelial bladder cancer (MIBC). However, loco-regional recurrence (LRR) is a common early event associated with poor prognosis. We evaluate 3-year LRR-free (LRRFS), metastasis-free (MFS) and overall survivals (OS) after adjuvant radiotherapy (RT) for pathological high-risk MIBC.

**Material and methods:**

We retrospectively reviewed data from patients in 3 institutions. Inclusion criteria were MIBC, histologically-proven urothelial carcinoma treated by RC and adjuvant RT. Patients with conservative surgery were excluded. Outcomes were evaluated by Kaplan-Meier method. Acute toxicities were recorded according to CTCAE V4.0 scale.

**Results:**

Between 2000 and 2013, 57 patients [median age 66.3 years (45–84)] were included. Post-operative pathological staging was ≤pT2, pT3 and pT4 in 16%, 44%, and 39%, respectively. PLND revealed 28% pN0, 26% pN1 and 42% pN2. Median number of lymph-nodes retrieved was 10 (2–33). Forty-eight patients (84%) received platin-based chemotherapy. For RT, clinical target volume 1 (CTV 1) encompassed pelvic lymph nodes for all patients. CTV 1 also included cystectomy bed for 37 patients (65%). CTV 1 median dose was 45 Gy (4–50). A boost of 16 Gy (5–22), corresponding to CTV 2, was administered for 30 patients, depending on pathological features. One third of patients received intensity-modulated RT. With median follow-up of 40.4 months, 8 patients (14%) had LRR. Three-year LRRFS, MFS and OS were 45% (95%CI 30–60), 37% (95%CI 24–51) and 49% (95%CI 33–63), respectively. Five (9%) patients had acute grade ≥3 toxicities (gastro-intestinal, genito-urinary and biological parameters). One patient died with intestinal fistula in a septic context.

**Conclusions:**

Because of poor prognosis, an effective post-operative standard of care is needed for pathological high-risk MIBC. Adjuvant RT is feasible and may have oncological benefits. Prospective trials evaluating this approach with current RT techniques should be undertaken.

## Introduction

Muscle-invasive urothelial bladder cancer (MIBC) (cT2-T4) is an aggressive disease with poor 5-year overall survival (OS) of 50% [1, 2]. Current optimal management is based on radical cystectomy (RC) and pelvic lymph node dissection (LND), generally associated with pre-operative cisplatin-based chemotherapy. Despite the enthusiasm for chemotherapy, loco-regional recurrence (LRR) remains an early event that appears in 4 to 25% of cases [2, 3]. Prognosis after LRR appears poor with a possible impact on metastasis-free survival (MFS) [4] and OS [5].

After surgery, pathological evaluation allows for adjuvant treatment strategies targeting this LRR, based on relevant staging rather than initial clinical staging. In the 1980’s, peri-operative radiotherapy (RT) was explored, demonstrating benefits in terms of loco-regional control, but associated with significant gastro-intestinal (GI) toxicity. This toxicity, directly dependent on the RT techniques used at the time has limited the development of this approach [6]. The aim of this study is to provide up-to-date estimations of outcomes and toxicity for patients treated by RC and adjuvant RT for MIBC in terms of 3-year LRR-free survival (LRRFS), MFS and OS. We focus on acute toxicities, in particular for patients with neobladders.

## Materials and methods

### Patients and treatments

After Groupe d’Etude des Tumeurs Uro-Genitales (GETUG) and French Urologist Association (AFU) National Boards approval, we retrospectively reviewed data from patients treated between January 2000 and December 2013 with adjuvant RT after surgery for MIBC in 3 institutions. Inclusion criteria were: patients older than 18 years treated by RC and postoperative RT for histologically-proven muscle-invasive bladder cancer. Cystectomy included *en bloc* excision of the bladder, prostate and seminal vesicles in men and the uterus, ovaries, and anterior vagina in women. Anatomo-pathological features included were pure urothelial carcinoma or dominant urothelial carcinoma (>50%) with a mixture of other subtypes.

No patient had distant metastases on pre-operative imaging of the chest, abdomen, pelvis, and bones. Patients treated by conservative surgery or for different histological subtypes (small cell variants, pure adenocarcinoma or pure epidermoid carcinoma) were excluded, whereas patients who received peri-operative chemotherapy were included. RT was realized 4 to 12 weeks after surgery. In case of adjuvant chemotherapy, a delay of 4–8 weeks between the end of chemotherapy and RT was respected. Chemotherapy could be delivered concomitantly to RT. Chemotherapy protocols were chosen by the physicians and consisted of platin-based regimens. For the RT schedules, in each center, a pelvic clinical target volume (CTV) was defined according to the Radiation Therapy and Oncology Group (RTOG) Atlas [7]. There was no consensus for cystectomy bed volumes. Concerning dose level, no specific guidelines were available in the literature and prescription dose depended on individual physician choices. Regarding neobladders dose constraints, due to the lack of published data, median dose should be as low as possible. All peri-operative treatment decisions were validated in a multidisciplinary team meeting.

Data including demographic status, initial tumor and pathological specimen characteristics, surgical techniques (including bladder diversion and LND status), chemotherapy, RT schedules, and patient follow-up were extracted from each patient’s file by the same physician. Tumors were restaged according to the 2009 TNM classification. Imaging follow-up schedules and modalities were heterogeneous across patients and centers, but the following examinations were performed at least every six months: physical examinations, thoracic computed tomography (CT), urine cytology and CT or magnetic resonance imaging (MRI) of the abdomen and pelvis. Acute toxicities (GI, genitourinary (GU), blood counts) were evaluated according to the Common Terminology Criteria for Adverse Events (CTCAE) version 4.0 scale. Particular attention was paid to acute toxicity for patients with neobladders.

### Statistical methods

LRR-free survival, MFS and OS were estimated by the Kaplan-Meier method and are reported with their 95% confidence intervals (CI) at three years. LRR was defined as soft tissue and /or lymph node recurrence in the pelvis anywhere between the aortic bifurcation into the primary iliac arteries and the pelvic floor below. LRR was determined on the basis of imaging (computed tomography (CT) / MRI) demonstrating soft tissue or nodal recurrence at least 1cm on the shortest axis at the level of the primary iliac and up to 8 mm below. Recurrence above the aortic bifurcation or within the inguinal nodes was noted as distant metastasis. For LRR-free survival, LRR and death without LRR were considered as events. Only patients alive at last news without LRR were censored. Similarly for MFS, distant metastasis and death of any cause were considered as events. Time to recurrence (loco-regional or distant) was calculated from the date of diagnosis to the date of event or death of any cause. OS was computed from the date of diagnosis to the date of death of any cause or last follow-up. All statistical analyses were performed by Stata V11.0. According to recent publications validating a LRR risk model [2, 8–12], we reported LRR rates in our population according to three risk levels: low, intermediate or high. Patients with limited MIBC (≤ pT2) were classified as low-risk. Patients with stages ≥ pT3, with extended LND (≥10 nodes removed) and good surgical margins (R0) were stratified as intermediate risk. Patients with disease ≥ pT3, with limited LND (<10 nodes removed) or involved surgical margins (R1), were classified as high LRR risk [12].

## Results

### Patients

Across the three centers, 57 patients were included and the median age was 66.3 years (range: 45–84). Patient’s characteristics are presented in Table 1.

**Table 1:** Clinico-pathological data and perioperative chemotherapy schedules for patients treated by radical cystectomy and adjuvant radiotherapy.

Parameters	Patients N (%)
Median age at diagnosis in years (range)	66.3 (45–84)
ECOG PS*	
0	21 (36.8)
1	35 (61.4)
2	1 (1.8)
Male	47 (82.5)
Female	10 (17.5)
Stage T TURB	
T1	4 (7)
T2	47 (82.5)
T3	2 (3.5)
T4	4 (7)
Stage N before radical cystectomy	
N0	39 (68.4)
N1	4 (7)
N2	1 (1.8)
N3	1 (1.8)
Nx	12 (21.1)
Multifocal lesions at TURB	
Yes	6 (10.5)
No	39 (68.4)
Unknown	12 (21.1)
Association with carcinoma in situ at TURB	
Yes	7 (12.3)
No	30 (52.6)
Unknown	20 (35.1)
Adjuvant CT regimen	27 (47.4)
GC	9 (15.8)
GCb	5 (8.8)
SD-MVAC	
Median number delivered cycles (SD)	6 (1.02)
Neoadjuvant CT regimen, No (%)	
DD-MAVC	2 (3.5)
SD-MVAC	2 (3.5)
GC	3 (5.3)
Median number of delivered cycles (SD)	4 (0.64)

*Eastern Cooperative Oncology Group (*ECOG); PS*: *Performance status; TURB*: *Transurethral resection of bladder; CT*: *chemotherapy; GC*: *gemcitabine-cisplatin; GCb*: *gemcitabine-carboplatin; MVAC*: *Methotrexate-vinblastine-adriamycine-cisplatin; DD*: *dose-dense; SD*: *standard; RC*: *radical cystectomy; SD*: *standard deviation*

### Surgery and peri-operative chemotherapy

Forty-eight (84.2%) patients received perioperative chemotherapy. Neoadjuvant and adjuvant schedules are detailed in Table 1. Pelvic LND was performed for 55 patients. One of them also had a para-aortic dissection. A neobladder was realized for 12 (21%) patients, while Bricker ileal conduits were created for 43 (75%) patients. For urinary diversion, one patient presented uretero-sigmoidostomy. Tumors were classified ≤pT2, pT3 and pT4 for 10 (17.5%), 25 (43.9%) and 22 (38.6%) patients, respectively. With a median number of 10 nodes retrieved (range: 2–33), lymph nodes were involved in 39 (68.4%) patients with a median of 2.33 involved nodes (range: 0–20). Surgical margins were R0 for 42 (73.7%) patients and R1 for 15 (26.3%). All pathological data are summarized in Table 2. Eleven patients (19%) were classified as low LRR risk, 15 (26%) intermediate and 30 (53%) high risk. One patient was unclassifiable because of lack of data about LND.

**Table 2:** Pathological characteristics at radical cystectomy.

Parameters	Patients N(%)
**Cystectomy urinary diversion**	
Neobladder	12 (21.1)
Bricker	43 (75.4)
Others	1 (1.8)
Unknown	1 (1.8)
**Lymphadenectomy**	
Pelvic	54 (94.7)
Para-aortic	1 (1.8)
Not realized	2 (3.5)
**pT (or ypT) Stage**	
pT1	1 (1.8)
pT2	9 (15.8)
pT3	25 (43.9)
pT4	22 (38.6)
**pN Stage**	
pN0	16 (28.1)
pN1	15 (26.3)
pN2	24 (42.1)
Unknown	2 (3.5)
**Multifocal lesions**	
Yes	8 (14)
No	47 (82.5)
Unknown	2 (3.5)
**Association with in situ carcinoma**	
Yes	9 (15.8)
No	35 (61.4)
Unknown	13 (22.8)
**Surgical Margin status**	
R0	42 (73.7)
R1	15 (26.3)
**Lymphovascular involvement**	
Yes	29 (50.9)
No	16 (28.1)
Unknown	12 (21.1)
**Number of lymph nodes retrieved**	
Median (range)	10.0 (2–33)
**Number of lymph nodes involved**	
Median (range)	2.33 (0–20)
**Percentage of involved lymph nodes**	
Median (range)	24.7 (0–100)
**Capsular rupture of lymph node metastasis**	
Yes	17 (29.8)
No	30 (52.6)
Unknown	10 (17.5)

### Radiotherapy

Concerning adjuvant RT, a Clinical Target Volume (CTV 1) including the pelvic lymph nodes was defined for all patients. Thirty-seven (65%) patients were treated on cystectomy beds, including four patients with neobladder. Median total dose in those volumes was 45 Gray (Gy) (range 4–50). Dose per fraction was 1.8 Gy for 37 patients (65%) and 2 Gy for 20 patients (35%). A boost radiation dose corresponding to CTV 2 was delivered on pelvic lymph nodes for 22 (38.6%) patients and on cystectomy beds for eight (14%) patients. Median boost dose was 16 Gy (range 5–22). Thirty eight (66.7%) patients were treated by conformational three-dimensional RT (3DCRT) and 19 (33.3%) patients by Intensity-Modulated Radiation Therapy (IMRT). Finally, seven patients were treated with concomitant platin-based chemotherapy as summarized in Table 3.

**Table 3:** Radiotherapy characteristics.

Parameters	Patients N (%)
RT techniques	
3DCRT	38 (66.7)
IMRT	19 (33.3)
Median Total dose, Gy (range)	45 (4–50)
Dose per fraction, Gy	
1.8	37 (64.9)
2	20 (35.1)
Irradiated volume	
Pelvic lymph nodes	57 (100)
Bed of cystectomy	37 (64.9)
Boost radiation	
Pelvic lymph nodes	22 (38.6)
Bed of cystectomy	8 (14.0)
No boost	27 (47.4)
Median boost dose, Gy (range)	16 (5–22)
Concomitant chemotherapy	
Cisplatin	2 (3.5)
Carboplatin	5 (8.8)
Not realized	50 (87.7)
Median number of delivered cycles (range)	4 (1–7)

Gy: Gray; SD: standard deviation

### Oncological outcomes

**Fig 1:**
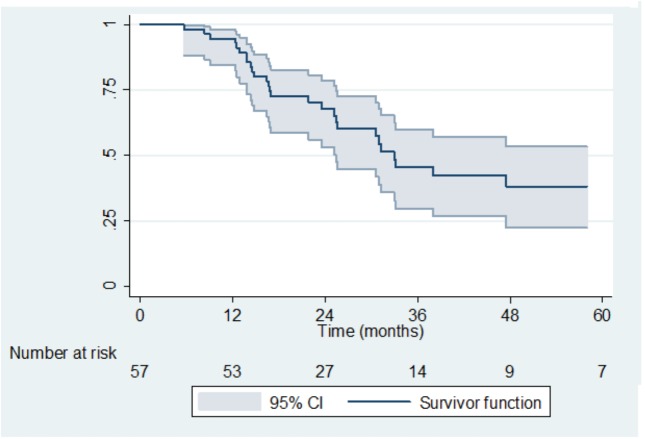
Loco-Regional Recurrence Free Survival.

**Fig 2:**
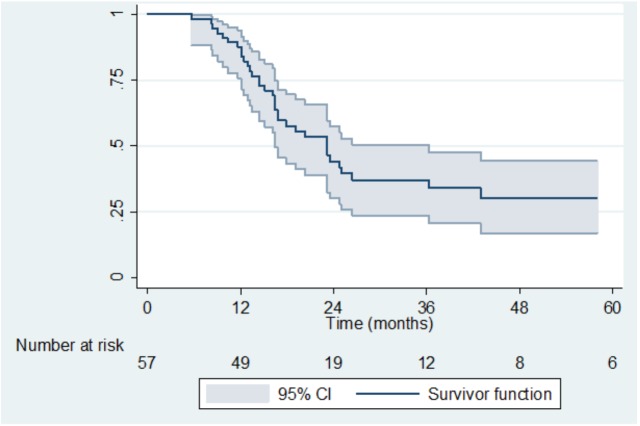
Metastasis Free Survival.

After a median follow-up of 40.4 months (range 26.1–62.4), 3-year LRR-free survival, MFS and OS were 45% (95%CI 30–60), 37% (95%CI 24–51) and 49% (95%CI 33–63), respectively (Figs 1 and 2). LRR occurred in eight patients (14%). According to the risk groups, LRR occurred for 1/11 (9%) low risk patients, 4/15 (27%) intermediate risk and 2/30 (7%) high risk patients. One patient with a LRR was unclassifiable according to risk stratification because of lack of data about LND.

LRR sites were pelvic lymph nodes for 6 patients including recto-sigmoid region, pre-sacral nodes and unspecified pelvic sidewall nodes for three patients. LRR encompassed multiple pelvic sites for three patients. For the six patients with pelvic lymph node relapse, regarding RC pathological staging, three presented pathological nodes at dissection, one had pathological margins, and one had limited LND with only 2 nodes retrieved. Two other patients had a single relapse in the cystectomy bed. These two patients were R0 and the cystectomy bed was not irradiated. For 4/8 (50%) patients, LRR treatment consisted of RT (N = 1), chemotherapy (N = 2) and radiofrequency (N = 1).

### Acute toxicities

Two patients (3.5%) had Grade 3–4 and 52 (91.2%) patients had Grade ≤2 Gastro-Intestinal (GI) toxicities. One patient died with intestinal fistula in a septic context (Grade 5). This patient has presented an abdominal pain with fever, five months after RT. Following imaging evaluation, an opacification of upper digestive tract has revealed fistula suspicion. That result has led to sepsis and death within one week. Regarding Genito-Urinary (GU) tolerance, two patients (3.5%) had Grade 3–4 toxicity and 52 (91.2%) presented Grade 1–2 toxicities With regard to biological parameters, renal disorder was observed in two patients (acute kidney injury with level of creatinine >3 x baseline or >4.0 mg/dL). Moreover, one patient treated with concomitant chemotherapy presented Grade 3 neutropenia. Finally, acute Grade ≥3 toxicities were observed in five patients (9%). Details of acute toxicities are reported in Table 4.

**Table 4:** Acute toxicities of adjuvant radiotherapy.

Adverse event	Patients N = 57
Gastrointestinal disorders	
Grade ≤ 2	52 (91.2)
Grade 3–4	2 (3.5)
Grade 5	1 (1.8)
Unknown	2 (3.5)
Urinary disorders	
Grade ≤ 2	52 (91.2)
Grade 3–4	2 (3.5)
Unknown	3 (5.3)
Renal disorders	
Grade ≤ 2	53 (93)
Grade 3–4	2 (3.5)
Unknown	2 (3.5)
Blood count disorders	
Grade ≤ 2	54 (94.7)
Grade 3–4	1 (1.8)
Unknown	2 (3.5)

### Patients with neobladder

Twelve patients had a neobladder. All of them were treated by adjuvant RT encompassing pelvic lymph nodes. Four patients were also irradiated on the cystectomy bed at a median total dose of 45 Gy (range 42–50.4). Five patients received a boost radiation (CTV 2) of 9 to 20 Gy, four in the pelvic lymph nodes, and one in the cystectomy bed. The RT technique was 3DCRT for seven patients and IMRT for five others. For the 12 patients with neobladder, two of them presented Grade ≥3 acute toxicities concerning GU and renal disorders. Additional Grade ≥3 hematologic adverse effects were reported for one of them. These two patients were treated with IMRT without cystectomy bed irradiation. One of them received a 10 Gy boost on one pelvic lymph node area (external iliac area). Dosimetric data, especially dose volume histogram, were only available for 6 patients. CTV encompassed pelvic lymph nodes for 5 patients and pelvic lymph nodes with cystectomy bed for one patient. For these 6 evaluable patients, neobladders prescribed dose could vary from 42 to 50.4 Gy. Two of them were treated with IMRT. Regarding dosimetric data on neobladders, median D2% was 48.2 Gy (range 37.5–56.2), median Dmax was 50.6 Gy (range 42.6–58) and median Dmean was 24.7 Gy (range 7.6–43.1).

## Discussion

Results from our study, with a medium-term follow-up in order to evaluate LRR, show that adjuvant RT for pathological high-risk MIBC is feasible and may have oncological benefits, especially since there is no other standard of care. We observed acceptable results in terms of acute toxicities. Indeed, less than 10% of patients presented grade > 3 toxicities although only 19 (33.3%) patients were irradiated with IMRT techniques. Some of them received dose escalation with boost that could impact acute toxicities. Moreover, our study included patients with neobladder and to our knowledge, there is no data regarding neobladder tolerance to RT. This study shows that orthotopic ileal neobladders can tolerate moderate doses of radiotherapy without significant induced morbidity but more data are required to provide important reassurance regarding the feasibility of including patients with orthotopic neobladders in studies examining the integration of surgery and post-operative radiotherapy. Regarding oncological outcomes, LRR was observed in 14% of cases with a median follow-up of 40.4 months. In our population, this rate may be considered as low, regarding the high-proportion of patients (26.3%) with positive surgical margins considered as the strongest independent predictive factor for LRR [1, 2, 13]. According to the risk stratification developed by the Philadelphia team [12], the expected rates of LRR were about 8%, 22% and 50% for low, intermediate and high risk groups, respectively. In our cohort, rates of LRR by subgroup were 9%, 27% and 7% respectively, clearly lower than expected for high risk patients and only for this subgroup. Even if we cannot show any correlation between LRR rates and RT in our study, this rate is in agreement with Zaghloul et al.’s results [6, 14] regarding the potential benefit of adjuvant RT, potentially reducing LRR rates by 50%.

### Adjuvant radiotherapy

Outcomes after adjuvant pelvic RT have been evaluated in a randomized trial with 236 patients with locally advanced bladder cancers, with only 20% of urothelial carcinomas [6]. Two different adjuvant RT schedules (conventional fractionation of 50 Gy in 2 Gy per fraction and hyper-fractionation of 37.5 Gy in 3 daily fractions of 1.25 Gy spaced every three hours), compared with cystectomy alone showed that adjuvant RT reduced pelvic failures (10%-13% vs. 50%, respectively for hyperfractionated and conventionally fractionated schedules). However, the rate of urothelial carcinomas, most commonly observed in Caucasian populations, mean that the LRR benefits can not necessarily be applied to patients with standard urothelial tumors as in our population.

Another prospective trial was performed with 100 patients comparing preoperative and postoperative pelvic RT (50 Gy, 2 Gy fraction). Approximatively half of patients presented urothelial carcinoma [15]. After a median follow-up of 32 months, in the postoperative RT arm, the 3-year OS, disease-free survival, LRRFS and MFS were 51.8%, 34.1%, 80.6%, and 55.7%, respectively. Despite these promising results, the approach was abandoned due to a significant rate of toxicities related to RT technique. In older studies [6, 14, 16, 17], the doses received by the rectum, the femoral heads, and especially the small intestine generated acute toxicities grade > 2. Recently, the development of 3DCRT, IMRT and imaging-guided radiation therapy (IGRT) has improved definition of CTVs and enabled sparing of adjacent organs-at-risk. Subsequently, many studies on pelvic RT have reported a decrease of irradiated intestinal volume with the use of modern techniques [18], including postoperative indications [19], highlighting a possible safe approach to investigate pelvic RT after cystectomy [20]. Our median dose of 45 Gy is relatively low compared to previous studies where 50 Gy were administered. However, several patients received a boost radiation and as such, a high dose of RT (> 60 Gy). This only applied to just over half of our patients, but it would explain the good loco-regional control despite a lower median total dose. Moreover, with modern techniques, we did not observe any additional toxicities despite these boost dose levels.

### Loco-regional recurrence rates

Surgical literature has traditionally reported LRR of 4–25% [3] including patients without any focus on a specific at-risk population unlike our study. Predictors of LRR, incidence, sites of recurrence and oncological impact are difficult to describe with accuracy [8]. LRR rates are inconsistently reported due to heterogeneous inclusion criteria in surgical studies, varying definitions of LRR and heterogeneity or lack of imaging follow-up protocols. Moreover, LRR is not always reported separately from distant relapse, and is commonly reported within the disease-free survival outcomes.

### Loco-regional recurrence predictive factors

Many studies have attempted to identify clinical and histological predictive factors of LRR [1, 2, 4, 13, 21–30]. However, the predictive role of these factors in multivariate analysis is controversial. Stein et al. [28] reported that 10-year OS decreased from 49 to 23% when lymph node involvement was present. Regarding the number of nodes removed during pelvic LND [29, 30], Herr et al. found a five-year OS of 61% when at least ten nodes were removed. OS decreased by 17% when less than ten nodes were removed supporting a curative role of pelvic LND [1]. The Southwest Oncology Group (SWOG)-Intergroup trial, randomizing neoadjuvant chemotherapy, showed a 32% LRR rate with ≥pT3 disease, a 29% rate with pN+ disease, and a 68% rate when margins were positive [1]. In Baumann et al.’s retrospective study [8] including 442 patients treated by RC and pelvic LND, with or without chemotherapy, stage of the disease (≥ pT3) (HR = 3.17) and limited dissection (<10 nodes removed) (HR = 2.37) were the only risk factors identified in multivariate analysis. Based on these results, Christodouleas et al. [2] included quality of resection to stratify patients regarding LRR risk. This risk model [2], validated on 2 different cohorts [10, 11], defined sub-populations at risk [2]. However, Baumann et al. [12] assessed the impact of multiple potential biases on the model’s validity reporting that these sources of bias did not invalidate the LRR risk stratification and did not change it. In mirror of our study, this competing risk multivariate analysis could improve accuracy to select patients most likely to benefit from improved local control with an adjuvant treatment.

### Loco-regional recurrence: Impact on survival

Loco-regional control has been demonstrated to have an impact on survival [4, 31–33]. Skinner et al. [31] reported higher loco-regional control rates at five years for patients who underwent an extensive LND than for those with limited dissection (85 vs. 63%). OS was improved if extensive LND was done, even in the absence of pelvic pathologic involvement (pN0). Other series have indicated a therapeutic role for extent of dissection and therefore loco-regional control of micrometastatic disease [5]. Furthermore, several studies have identified an association between loco-regional control and the occurrence of distant metachronous metastases [4, 33]. If a relationship exists between LRR and survival, adjuvant loco-regional therapy is needed. In this regard, it is important to stress that the administration of chemotherapy does not bring any significant benefits in terms of loco-regional control [1, 34–37]. Those key-points were the background to propose adjuvant RT to our patients.

### Schedule of adjuvant radiotherapy

Regarding recently published data concerning patterns of loco-regional failures and risk stratification [9, 12], in our population, adjuvant RT indications and irradiated volumes could appears questionable. However, all patents received pelvic radiation including common iliac regions, iliac internal and external and obturator nodes, as recommended. Concerning high risk patients, they did not all receive pre-sacral and cystectomy bed radiation, yet only two relapses in the cystectomy bed were observed. These two patients had safe surgical margins and were not considered as high risk, so did not receive any radiation in the cystectomy bed.

### Limitations

Some biases due to the retrospective design and the relatively small number of patients limit the relevance of our results. We could not validate Christodouleas et al.’s [2] stratification model due to the lack of events observed. In addition, our population was quite heterogeneous with low risk patients included in a potentially questionable RT schedule, even if all indications were validated in a multidisciplinary team meeting. It could be highlighted that most patients eligible for neoadjuvant chemotherapy did not receive this treatment, probably related to the long inclusion period, with practices evolving over time. This could explain our lower results about MFS and OS compared to the literature. Further, the RT schedules were quite heterogeneous, due to lack of consensus for technique, contouring, dose and patient indication. Based on mapping proposed by Baumann et al. [9] identifying preferential sites of pelvic LRR, an international consensus about contouring guidelines for adjuvant RT is currently in progress [38]. Finally, despite some recent promising local control results with adjuvant sandwich chemotherapy and radiation versus adjuvant chemotherapy alone for locally advanced bladder cancer, indications of concomitant chemotherapy proposed in our study was absolutely not consensual [39].

## Conclusions

In this retrospective contemporary cohort including only standard urothelial bladder cancers treated by RC, adjuvant RT showed good loco-regional control. Tolerance was acceptable (less than 10% of grade ≥ 3 toxicities). With postoperative nomograms correlating tumor pathological characteristics (pT, pN, number of lymph nodes retrieved, margin status) with LRR incidence and preferential sites, it could be possible to target more accurately the “at-risk” areas for well selected patients with adjuvant RT. Moreover, technical considerations such as use of IMRT and IGRT in this indication may allow lower toxicities by better sparing of adjacent organs-at-risk. For patients with pathological-risk MIBC, no postoperative standard of care is established despite the poor prognosis. Several groups such as the National Cancer Institute in Egypt, groups in North America (NRG Oncology), France (GETUG-AFU), United Kingdom (NCRI) and India (Tata Memorial Hospital) have already opened or are in the process of developing phase II trials to re-evaluate the feasibility of adjuvant RT for MIBC.
